# Functional Coherence in Intrinsic Frontal Executive Networks Predicts Cognitive Impairments in Alcohol Use Disorder

**DOI:** 10.3390/brainsci13010045

**Published:** 2022-12-26

**Authors:** Nicola Canessa, Gianpaolo Basso, Marina Manera, Paolo Poggi, Claudia Gianelli

**Affiliations:** 1 IUSS Cognitive Neuroscience (ICON) Center, Scuola Universitaria Superiore IUSS, 27100 Pavia, Italy; 2 Istituti Clinici Scientifici Maugeri IRCCS, Cognitive Neuroscience Laboratory of Pavia Institute, 27100 Pavia, Italy; 3 School of Medicine and Surgery, University of Milano-Bicocca, 20126 Milan, Italy; 4 Istituti Clinici Scientifici Maugeri IRCCS, Clinical Psychology Unit of Pavia Institute, 27100 Pavia, Italy; 5 Istituti Clinici Scientifici Maugeri IRCCS, Radiology Unit of Pavia Institute, 27100 Pavia, Italy; 6 Department of Clinical and Experimental Medicine, University of Messina, 98122 Messina, Italy

**Keywords:** alcohol use disorder, intrinsic brain activity, *f*MRI, executive network, salience network, compensatory mechanisms, fronto-lateral cortex, default-mode network, rehabilitation

## Abstract

Growing evidence highlights the potential of innovative rehabilitative interventions such as cognitive remediation and neuromodulation, aimed at reducing relapses in Alcohol Use Disorder (AUD). Enhancing their effectiveness requires a thorough description of the neural correlates of cognitive alterations in AUD. Past related attempts, however, were limited by the focus on selected neuro-cognitive variables. We aimed to fill this gap by combining, in 22 AUD patients and 18 controls, an extensive neuro-cognitive evaluation and metrics of intrinsic connectivity as highlighted by resting-state brain activity. We addressed an inherent property of intrinsic activity such as intra-network coherence, the temporal correlation of the slow synchronous fluctuations within resting-state networks, representing an early biomarker of alterations in the functional brain architecture underlying cognitive functioning. AUD patients displayed executive impairments involving working-memory, attention and visuomotor speed, reflecting abnormal coherence of activity and grey matter atrophy within default mode, in addition to the attentional and the executive networks. The stronger relationship between fronto-lateral coherent activity and executive performance in patients than controls highlighted possible compensatory mechanisms counterbalancing the decreased functionality of networks driving the switch from automatic to controlled behavior. These results provide novel insights into AUD patients’ cognitive impairments, their neural bases, and possible targets of rehabilitative interventions.

## 1. Introduction

Executive functioning refers to cognitive processes underlying the self-regulation of goal-directed actions, and thus the ability to avoid maladaptive behaviors, via top-down inhibitory control on bottom-up appetitive drives [1,2]. Defective executive skills are indeed considered a core facet of cognitive alterations in addictions, including alcohol use disorder (AUD) [2,3,4]. The chronic compulsive drinking behavior inherent in AUD, leading to over 3 million deaths per year (WHO), is indeed associated with negative emotional states during abstinence and, therefore, loss of control over alcohol intake.

As for other addictions, maladaptive behavioral learning might promote the establishment of drinking habits [5,6], which are reinforced by the reward inherent in consumption (positive reinforcement) and/or in avoiding the punishing experience associated with abstinence (negative reinforcement) [7]. The latter, in particular, might drive the progression from impulsivity to compulsivity by potentiating the avoidance of craving-related aversive states. At the neural level, this process seems to involve a progressive imbalance between reward-related “reflexive” drives generated by the limbic subcortical structure and “reflective” skills of executive control associated with fronto-striatal networks, mediating the shift from goal-directed to habitual behaviors [8]. Increasing evidence indeed shows prominent executive deficits in AUD [2,9], reflecting structural and/or functional alterations in fronto-striatal networks underlying executive control [10].

The presence of such impairments in AUD has several consequences. First, these changes might promote a vicious circle in which altered decision-making mediates the effect of decreased executive skills on relapses, thus further impairing inhibitory skills and perpetuating addictive behaviors. Cognitive remediation procedures increase the likelihood of breaking this circle, with cognitive enhancements being transferred to functional improvements [11], but again treatment efficacy depends on “baseline” executive skills [12]. Moreover, despite the promising effects of neuromodulation techniques on craving [13,14] and cognitive control [15] in alcoholic patients, this approach requires the preliminary identification of “target” regions.

This multifaceted evidence highlights the need for a detailed characterization of executive impairments, and their neural bases, in AUD. By coupling an extensive neuro-cognitive assessment with magnetic resonance imaging (MRI) metrics of brain structure and function, we have recently addressed this issue via voxel based morphometry (VBM) [16,17,18], diffusion tensor imaging (DTI) [19,20], and resting-state functional magnetic resonance imaging (rs-*f*MRI) [21,22,23]. The latter approach is becoming increasingly valuable in clinical neuroscience, based on the spatial overlap between patterns of brain activity underlying task performance and the networks showing low-frequency and temporally coherent fluctuations of the Blood Oxygen Level Dependent (BOLD) signal at rest [24]. Variations of performance across several domains have been related to distinct metrics of such a resting state, i.e., “intrinsic” brain activity, both in normal subjects [25] and pathological populations [26]. In AUD, a prominent impairment of basic executive functions such as working memory, attention and visuomotor speed reflects grey matter (GM) loss [16,17], as well as abnormal intrinsic connectivity [19,20,21,22,23], in fronto-striatal and fronto-insular regions mediating the recruitment of controlled processes by salient stimuli.

We aimed to complement these findings by focusing on an inherent property of intrinsic brain activity such as the degree of resting-state *synchrony*. Building on our previous findings from the same dataset [21], we here assessed the temporal correlation of intra-network BOLD fluctuations within sets of areas associated with behavioral control, e.g., executive, salience, attentional and default-mode (DMN) networks. Previous related studies have provided inconsistent data in this respect, i.e., extensive [27,28,29], focal [30,31] or even absent [32] alterations of intra-network connectivity in AUD. Moreover, due to a prominent interest towards specific cognitive functions, most previous studies lacked comprehensive neuro-cognitive evaluations [29,31,33]. We aimed at filling this gap by coupling an extensive neuro-cognitive assessment with the analysis of intra-network coherence which, compared with other metrics of resting-state networks, represents an earlier biomarker of pathological changes in the intrinsic functional brain architecture [34].

On the basis of our previous data [16,17,18,19,20,21,22,23], we expected AUD patients’ executive impairment to be reflected in abnormal internal coherent activity and GM atrophy in networks underpinning the interplay between automatic and controlled activity and processing, such as default mode, attentional, salience and executive networks.

## 2. Materials and Methods

### 2.1. Participants

We recruited 22 alcoholic patients and 19 control participants matched according to age, education and gender distribution (Table 1). Patients joined the study during inpatient alcohol withdrawal treatment. They underwent MRI scanning when being detoxified for at least 10 days, and at least 8 days after benzodiazepine treatment. Patients were enrolled based on the following criteria: (1) AUD diagnosis based on DSM-5; and (2) aged between 20 and 60. Exclusion criteria for patients and controls were: (1) current/past neurological or psychiatric disorders except for AUD, or comorbid disorders except for tobacco use disorder; (2) family history of neurological or psychiatric disorders; (3) ongoing intake of psychotropic substances/drugs; (4) past cerebral damage and/or loss of consciousness; (5) severe medical conditions (e.g., malnutrition, diabetes, liver or kidney diseases); and (6) incompatibility with MRI. Controls were also excluded in case of history or presence of alcohol abuse, after a screening based on mean alcohol intake <2 and 1 Alcohol Units (UA) for, respectively, males and females (1 UA = 12 g of ethanol, e.g., 330 mL beer, 125 mL wine, or 40 mL hard liquor). Controls were also requested to remain abstinent for a minimum of 10 days before the neuroimaging session.

Participants provided written informed consent, and the study was previously approved by the ICS Maugeri Ethical Committee. The study was performed according to the most recent version of the Declaration of Helsinki.

The top table section reports the mean and standard deviation (SD) of demographic information and smoking status for patients (AUD) and healthy controls (HC), along with results from group comparisons based on 2-sample t-tests and a chi-square test. The bottom table section reports (separately for males and females) the duration of alcohol use and mean daily intake (standard alcohol units, UA), along with results of gender comparisons from two-sample *t*-tests.

### 2.2. Neuropsychological Assessment

#### 2.2.1. Neuropsychological Battery

We used the Brief neuropsychological examination (ENB2) [35] to assess the following cognitive domains: attention (trail making test A and B), memory (immediate/delayed recall; digit span), working-memory (interference-memory at 10/30 seconds), executive skills (trail making test B, phonemic fluency, abstract verbal reasoning, overlapping pictures, cognitive estimation and clock-drawing), plus perceptual and praxis skills (spontaneous and copy drawing tasks; ideative and ideomotor praxis test). The single-task scores allowed for the computing of domain-specific and total cognitive scores.

#### 2.2.2. Analysis of Neuro-Cognitive Performance

Due to the drop-out of one control participant, statistical analyses included 22 patients and 18 controls. The details on neuropsychological analyses have been previously reported [16,21]. We took a multivariate approach based on a principal component analysis on neuropsychological scores to investigate patients’ cognitive impairments in terms of higher-level domains cutting across single tasks (Supplementary Tables S1–S3 and Supplementary Figure S2). The resulting individual factor scores were used to assess possible differences in performance across groups via an analysis of variance (ANOVA). Statistics were Bonferroni corrected for the number of tests performed, and ancillary analyses were carried out to control for the potential effect of smoking status.

### 2.3. Resting-State fMRI Study

#### 2.3.1. Acquisition of MRI Data

We collected (*f*)MRI data with a General Electrics (GE Healthcare, Milwaukee, Wisconsin, USA) 3Tesla MR750 scanner. The resting-state *f*MRI scan lasted 8 min, corresponding to 240 volumes each including 37 ascending continuous axial sections with Time-to-Repeat (TR) = 2000 ms, Echo Time (TE) = 30 ms, flip-angle = 78°, Field-of-View (FoV) = 19.2 cm, matrix = 64 × 64, thickness = 4 mm, and gap = 0.2 mm. Participants were instructed to fixate a crosshair during the resting-state *f*MRI scan [36]. We additionally collected an anatomical T1-weighted IR-prepared FSPGR (BRAVO) scan (152 sections, resolution = 1 mm^3^).

#### 2.3.2. RS-fMRI Data Pre-Processing

We used SPM12 (http://www.fil.ion.ucl.ac.uk/spm, accessed on 5 May 2022) to perform a pre-processing of *f*MRI data that was corrected for slice-dependent delays, spatially realigned to the first volume and unwarped, spatially normalized to the standard MNI (Montreal Neurological Institute) space [37] and resampled (2 mm^3^ voxels), and spatially smoothed (8 mm full-width half-maximum (FWHM) isotropic Gaussian kernel). Finally, the Motion Fingerprint toolbox [38] was used to compute a comprehensive motion index based on the SPM realignment parameters. In two patients, few isolated volumes with large (>2 mm) scan-to-scan head motion were removed by interpolation, resulting in no significant group differences in framewise displacement.

The GIFT toolbox (http://icatb.sourceforge.net, accessed on 3 June 2022) was then used to perform a group independent component analysis (gICA), extracting 75 temporally coherent and spatially independent sources, i.e., “spatial maps” reflecting functional networks, from the whole sample rs-*f*MRI timecourses. In line with well-established recommendations [39], we selected resting-state networks (RSNs) using (a) the spectral characteristics of component timecourses; (b) a stability index (Iq) reflecting the consistency of the differential intra/extra-cluster similarity across 250 ICA rounds [40]; and (c) a visual inspection of spatial maps. The 57 retained maps (Supplementary Figure S1) were anatomically labelled in terms of resting-state networks with the GIFT RNS template, and in terms of anatomical regions with the Anatomy toolbox v2.239 (Supplementary Tables S4–S7) [41].

#### 2.3.3. RS-fMRI Statistical Analyses

Our dependent variable within the GIFT framework was represented by the spectral power of RSN timecourse, reflecting the contribution of single frequency bins to intrinsic BOLD fluctuations, and therefore the degree of coherence in intra-network activity (maximal for high power spectra at low frequencies) [39].

We used two-sample *t*-tests, multiple regressions and task-by-group interactions to identify the frequency bins in which spectral power reflected, respectively: (a) group differences, (b) a relationship with cognitive performance in the domain(s) showing the strongest impairment in patients, or (c) an interaction between the group and this relationship (i.e., a significantly different regression slope across groups). Based on behavioral results (3.1), we modelled the basic-level executive factor associated with interference-memory and TMTa tasks. Univariate tests were performed, modelling group (AUD/controls), performance and performance-by-group interaction as factors, plus nuisance variables coding head motion [42,43], age, smoking status and total intracranial volume. This analysis was aimed to isolate spectral bins specifically associated with cognitive performance, group, or their interaction, while removing the potential effects of such nuisance variables. Ancillary analyses were performed to test a link between the amount of alcohol consumption and spectral power in the frequency bins showing significant effects in the main analysis.

Statistics were thresholded at *p* < 0.05, corrected for multiple comparisons based on False Discovery Rate (FDR) [44].

### 2.4. VBM Pre-Processing and Statistical Analyses

To assess a relationship between structural and intrinsic functional alterations in AUD, we first used the Computational Anatomy Toolbox (CAT12; www.neuro.uni-jena.de/cat/, accessed on 3 June 2022) to perform a pre-processing of anatomical MRI images [45] including: (a) bias correction (to correct non-uniformities in voxel intensity); (b) spatial normalization (to warp individual images to the MNI space); (c) segmentation (to extract GM); and (d) smoothing with an isotropic 8 mm FWHM gaussian kernel (to reduce inter-individual variation).

In subsequent statistical analyses, we first assessed group differences in GM concentration in the RSNs showing the above effects of interest. For each RSN mask we used a two-sample t-test to compare mean GM concentration across groups (*p* < 0.05 FDR corrected). On the basis of the previously reported relationship between executive performance and both GM atrophy and intrinsic connectivity in the fronto-lateral cortex [16,21], we then assessed whether GM concentration in the RSN maximally encompassing this region mediates the relationship between its degree of coherent activity and cognitive performance. We carried out this analysis on the “executive” component 43, both because it encompassed our region of interest in the fronto-lateral cortex, and because interaction analyses highlighted a significantly stronger relationship, in patients compared with controls, between its degree of coherent activity and executive performance (3.2.2).

“Mediation” entails that the relationship between the dependent and independent variables represents an indirect effect due to a mediator variable. In this case, including the mediator in a regression model along with the independent variable is expected to reduce the effect of the latter, while leaving a significant effect of the former. Therefore, the hypothesis that GM concentration in component 43 mediates the effect of its coherent activity on executive performance was assessed through the Sobel-Goodman test [46] based on the following criteria: (a) an association between mediator and independent variable; (b) an association between dependent and independent variables in the lack of the mediator; (c) a significant unique effect of the mediator on the dependent variable; and (d) a significant decrease of the effect of the independent variable on the dependent variable when the mediator is included in the model.

## 3. Results

### 3.1. Neuro-Cognitive Performance

There was no significant group difference regarding age or educational level, but the proportion of smokers was significantly higher among patients than controls (Table 1). This potential confounding variable was therefore considered in subsequent analyses. A principal component analysis reduced the full dataset of ENB2 scores to six components which explained 76.42% of their variance (Supplementary Table S8 [41]), involving basic-level and high-level executive processes, verbal learning visual-constructional abilities, language, and cognitive estimation (Supplementary Table S9) [16,21]. A significant group difference for the score of the third component (F(1,38) = 12.15, *p* < 0.005) highlighted worse executive performance in patients than controls, as indexed by its contributing attentional (TMT-A; *r* = −0.79, *p* < 0.001) and working-memory (interference-memory; *r* = 0.72, *p* < 0.001) scores. We therefore modelled this factor score in subsequent analyses on intrinsic brain activity and GM concentration.

### 3.2. Neuroimaging Results

#### 3.2.1. Intrinsic Brain Activity

Both a visual inspection of the spatial maps generated by gICA and the analyses of their spectral characteristics led to the retaining of 57 components involving all the main RSNs [21,39] (Supplementary Figure S1 and Supplementary Tables S4–S7 [41]).

#### 3.2.2. RS-*f*MRI Results: Spectral Power and Level of Intra-Network Coherent Activity

**Figure 1:**
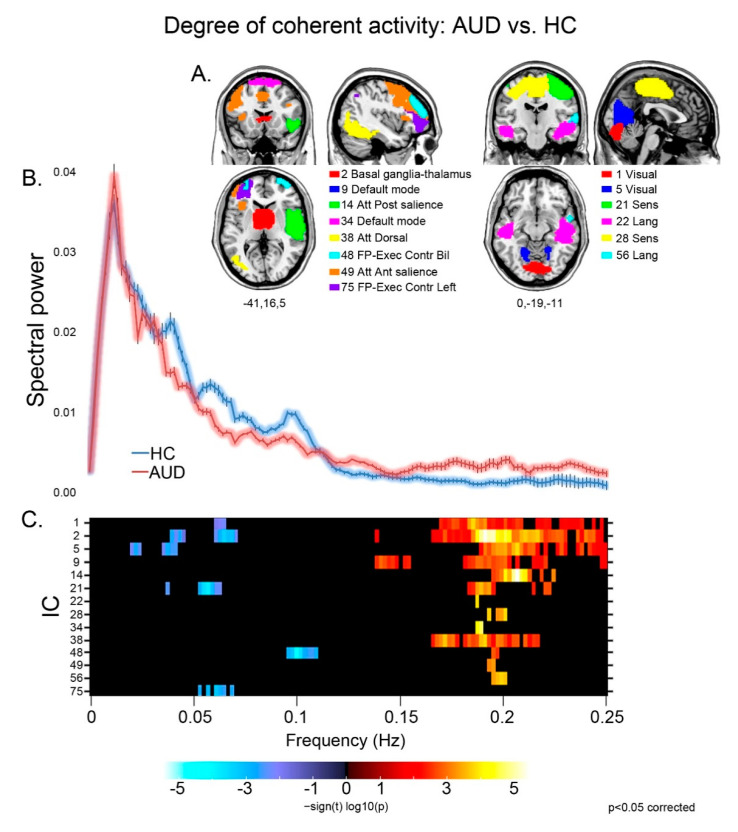
Degree of coherence of intra-network activity: group effects. For the components displaying a significant group difference (**A**), the strength and direction of this effect is shown by the colored frequency bins depicted in panel (**C**). In the latter, decreased coherence of intra-network activity is shown by reduced low frequency power (<0.1 Hz; blue), increase of high frequency power (>0.1 Hz; red), or both. In panel (**B**), the plots of spectral power depict the grand average, across the significant components reported above, of mean (± standard error) spectral power in the 0–0.25 Hz frequency band for controls (blue) and patients (pink).

Patients displayed different aspects of faster BOLD fluctuations, suggestive of decreased coherence of intra-network activity compared with controls [39], in several networks (Figure 1). They displayed both decreased low (≤0.1 Hz) and very low (<0.05 Hz) frequency power, and increased high (>0.1 Hz) frequency power, in sensorimotor (21), visual (1,5), basal ganglia (2) and executive (48) networks. Other components displayed either decreased low frequency power in the left executive control network (75), or increased high frequency power in the default mode (9,34), sensorimotor (28), dorsal attentional (38) and language (22,56) networks, and in both the anterior (49) and posterior (14) sectors of the salience network.

**Figure 2:**
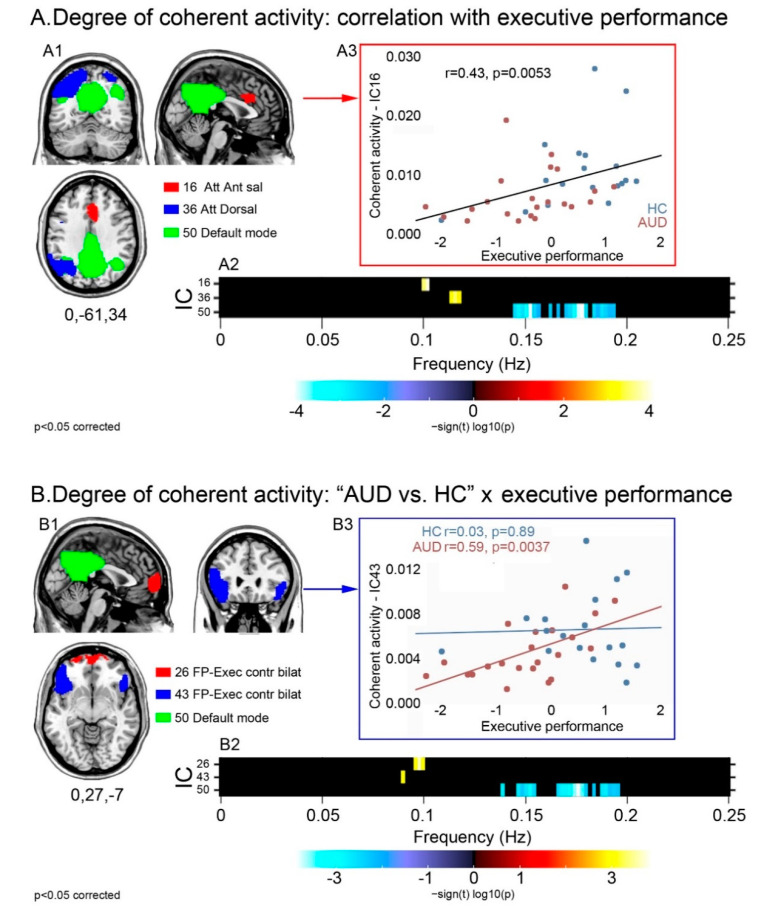
Degree of coherence of intra-network activity: relationship with executive skills in the whole sample and task-by-group interaction. For the components displaying a significant relationship with executive skills (**A1**), or a task-by-group interaction (**B1**), the strength and direction of this effect are shown by the frequency bins depicted in the respective panels (**A2**,**B2**). Regardless of group, the relationship between executive performance and intra-network coherent activity is depicted either by the positive correlation between performance and low frequency spectral power (higher coherent activity) in attentional and anterior salience networks, or by the negative correlation between performance and high frequency spectral power (lower coherent activity) in the posterior DMN (**A2**). Task-by-group interaction analyses highlighted a stronger relationship with executive performance, in patients than controls, in the same posterior DMN as well as in two frontal sectors of the executive control network (**B2**). Scatterplots show the correlation between executive performance and coherence of activity in the anterior salience network regardless of group (**A3**), and in the fronto-lateral executive network more in AUD patients than controls (**B3**).

Executive performance was positively correlated with power at the higher bound of low frequencies (=0.1 Hz) in the dorsal (36) and anterior salience (16) attentional networks, and negatively correlated with high frequency power in the posterior DMN (50) (Figure 2A). Spectral power did not differ across groups in these components. Instead, there was a significant task-by-group interaction in the executive (26,43) and posterior default mode (50) networks (Figure 2B): patients displayed a stronger relationship, compared with controls, between executive performance and (a) greater low frequency power in the executive components 26 and 43; and (b) smaller high frequency power in the posterior DMN (50). Again, none of these components displayed a significant main effect of group in spectral power.

Control analyses highlighted no significant relationship between mean daily alcohol dose and coherence of intra-network activity in the aforementioned components.

#### 3.2.3. VBM Results: Mediation Effect of GM Concentration

**Figure 3:**
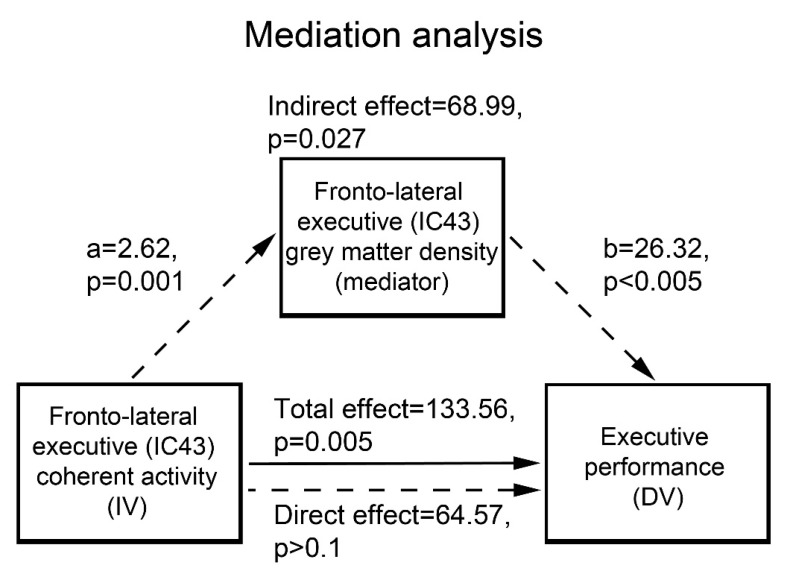
Mediation analysis.

GM concentration was significantly decreased, in patients vs. controls, in all the RSNs displaying the above effects of interest. In particular, 52% of the effect of fronto-lateral (component 43) coherent activity on executive performance was significantly mediated by its average GM concentration (*p* = 0.027) (Figure 3).

For the components displaying a significant group difference (A), the strength and direction of this effect is shown by the colored frequency bins depicted in panel C. Decreased coherence of intra-network activity is shown by reduced low frequency power (<0.1 Hz; blue), an increase of high frequency power (>0.1 Hz; red), or both. In panel B, the plots of spectral power depict the grand average, across the significant components reported above, of mean (± standard error) spectral power in the 0–0.25 Hz frequency band for controls (blue) and patients (pink).

For the components displaying a significant relationship with executive skills (A1), or a task-by-group interaction (B1), the strength and direction of this effect are shown by the frequency bins depicted in the respective panels A2–B2. Regardless of group, the relationship between executive performance and intra-network coherent activity is depicted either by the positive correlation between performance and low frequency spectral power (higher coherent activity) in attentional and anterior salience networks, or by the negative correlation between performance and high frequency spectral power (lower coherent activity) in the posterior DMN (A2). Task-by-group interaction analyses highlighted a stronger relationship with executive performance, in patients than controls, in the same posterior DMN as well as in two frontal sectors of the executive control network (B2). Scatterplots show the correlation between executive performance and coherence of activity in the anterior salience network regardless of group (A3), and in the fronto-lateral executive network more in AUD patients than controls (B3).

Average GM concentration in the fronto-lateral sector of the executive network (component 43) mediates 52% of the effect of its coherent activity on executive performance. IC: independent component; IV: independent variable; DV: dependent variable.

## 4. Discussion

We have previously described a relationship, in AUD, between executive deficits and altered connectivity among the fronto-insular-striatal nodes of the “control” networks [21]. Here we enriched those data by assessing a core facet of intrinsic brain connectivity such as intra-network coherence, which, compared with other resting-state metrics, represents an earlier biomarker of pathological changes in the intrinsic functional brain architecture [34]. Unlike most previous studies on this topic, we pursued this aim through a multivariate approach to the analysis of both neuro-cognitive and resting-state *f*MRI data to detect intrinsic networks where altered functional coherence relates to AUD patients’ defective performance in cognitive domains transcending single tasks.

This approach unveiled a strongly impaired executive domain in AUD patients, with defective performance on TMTa and interference-memory tasks signalling decreased visuomotor speed, attention and working-memory (Supplementary Table S9) [16,21]. In line with the notion that cognitive impairments in AUD reflect widespread neuro-functional changes involving subcortical nodes [47], the analysis of spectral power revealed decreased intra-network coherent activity, in patients vs. controls, within several components involving the default mode, executive, attentional and striatal brain networks (Figure 1). Such a shift to faster BOLD fluctuations, previously reported in healthy ageing [39], is a typical hallmark of neurological [26] and psychiatric [48,49,50] conditions, reflecting the dysregulation of cortical activity and associated cognitive functioning [51]. Since slow fluctuations are considered to underpin temporal synchronicity between regions underlying homologous functions, a decrease of internal coherence might track altered connectivity between and within networks [50] and, accordingly, of neural processes underlying cognitive performance.

The present findings therefore contribute to a literature debate reporting either absent [32], selective [30,31] or widespread [27,28,29] alterations of intra-network functional connectivity in AUD. Consistent with the hypothesis that cognitive impairments in AUD reflect more of a global than a selective pattern of brain alteration [47], all of these components also displayed GM atrophy in patients. In keeping with the close relationship between the structural and intrinsic functional brain architecture [52], intra-network temporal coherence could therefore highlight further cues into the brain correlates of defective cognitive performance, and/or compensatory mechanisms, in AUD. We addressed this issue with correlation and interaction analyses, to evaluate quantitative vs. qualitative group differences in the association between intra-network coherent activity and executive performance, respectively.

In both groups, higher executive scores were reflected in slower BOLD fluctuations in the anterior salience (16), dorsal attentional (36), and posterior default mode (50) networks (Figure 2A), all supporting cognitive performance proportionally to the level of preservation of their internal coherent activity. While GM concentration was significantly reduced in AUD patients in all of these components, none were associated with a significant decrease of coherence in intrinsic activity, which suggests that its degree reflects individual differences in executive skills along the continuum between normal and impaired performance. This interpretation fits with the role of these networks in driving the interplay between automatic and controlled cognitive processing when salient external stimuli are detected [53]. This switch is indeed prompted by the salience network, including the anterior insula, dorsal anterior cingulate cortex (dACC), basal ganglia and thalamus [54], which activates the attentional and executive control networks to recruit resources for controlled processing [55]. This switch entails the suppression of DMN activity to maintain cognitive sets and manipulate information in working-memory when focusing attention on task-relevant goals [56]. Indeed, while stronger DMN suppression is related to enhanced cognitive performance in normal conditions [57,58], an impairment of this mechanism has been shown, in alcoholic patients, by task-related *f*MRI evidence [59]. Decreased functional coherence in the anterior salience, dorsal attentional and default mode networks, tracking the degree of executive dysfunction, might thus reflect an abnormal interplay, in AUDs, between anti-correlated neural systems underlying the switch from automatic to controlled processes. This notion complements previous evidence of altered connectivity, in alcoholic patients, between the two key “salience” nodes in the insula and dACC [60].

Compared with controls, patients also exhibited a stronger relationship between coherence of activity and executive performance in two distinct sectors of the frontal control network involving the medial frontopolar and bilateral fronto-lateral regions alongside the posterior DMN. As shown by the representative scatterplot depicted in Figure 2B, this *qualitative* group difference reflects a relationship between cognitive performance and coherent activity in the frontal executive control network that is unique to patients. This finding might thus highlight a compensatory mechanism in AUD, whereby performance reflects the effectiveness of residual frontal executive neural mechanisms counterbalancing the decreased functionality of the salience-based engagement of controlled processes. The possible connection between the functional and structural bases of this mechanism is supported by the present evidence that GM concentration in this frontal component-significantly decreased in AUD patients, mediating 52% of the effect of its coherent activity on executive performance (Figure 3). In line with the previously reported effects of fronto-lateral stimulation on executive performance [61] and craving [13,14], these results highlight this region as a promising target of neuro-stimulation treatments aiming at enhancing executive skills in addictions.

There are possible limitations to this study. First, the small-to-moderate size of the patient and control groups indicates that these findings require supporting evidence from studies based on larger samples. Moreover, modelling smoking status in statistical analyses might remove only part of the contribution of nicotine consumption to the reported group differences. Finally, the present evidence of an association between impaired executive skills and altered intrinsic neural functioning would benefit from the inclusion of tasks assessing the inhibition of prepotent habitual responses. Notwithstanding these limitations, the present evidence paves the way to future extensions addressing the neural bases of defective executive functioning in AUD and their susceptibility to treatment in greater depth

## 5. Conclusions

The present findings support and extend our previous evidence of abnormal inter-network intrinsic connectivity underlying executive deficits in alcoholic patients [21,22,23]. By showing that GM atrophy in fronto-lateral regions mediates the relationship between their degree of internal coherent activity and executive performance, these data shed light on the multi-modal neural correlates of cognitive impairment in AUD. In line with our previous morphometric evidence [16], these data suggest that, in this condition, decreased executive performance reflects an abnormal interplay within the networks mediating the ability of salient stimuli to drive the switch from automatic to controlled processing, i.e., from default-mode to executive modes. These results provide several cues for future research, such as assessing the relationship between subtler cognitive alterations in AUD [62] and other facets of brain connectivity, as with white-matter organization, or multivariate features combining the MRI and EEG [63] modalities.

## Figures and Tables

**Table 1:** Demographic and alcohol intake variables.

Demographic Variables (Controls and Patients)	Age (Years)	Education (Years)	Smoking
Controls: Mean (SD)	45.11 (8.69)	10.11 (2.78)	6 out of 19
Patients: Mean (SD)	45.56 (7.99)	9.91 (2.65)	18 out of 22
*p*-value	0.426	0.405	<0.01
Alcohol use variables (patients only)	Duration of alcohol use (years)	Mean daily alcohol dose	Past use of other substances
Females: Mean (SD)	11.89 (7.11)	14.94 (5.92)	None
Males: Mean (SD)	10.11(7.48)	14.18 (7.12)	Marijuana (*n* = 1); cocaine and marijuana (*n* = 2)
*p*-value	0.576	0.791	

## Data Availability

The datasets generated during and/or analysed during the current study are available from the corresponding author on reasonable request.

## Supplementary Materials

The following supporting information can be downloaded at: https://www.mdpi.com/article/10.3390/brainsci13010045/s1, Figure S1: Resting-state networks, Figure S2: Scree plot of the principal component analysis performed on the 15 scores of the Brief Neuropsychological Examination (ENB2), Table S1: Correlation Matrix, Table S2: Communalities, Table S3: Rotated component matrix, Table S4: Peak activations of default-mode and executive networks, Table S5: Peak activations of attentional networks, Table S6: Peak activations of language, sensorimotor, visual and auditory networks, Table S7: Peak activations of limbic, basal ganglia and cerebellar networks, Table S8: Total variance explained, Table S9: Principal component analysis of neuro-cognitive data.
