# Supplementary material for: Functional Coherence in Intrinsic Frontal Executive Networks Predicts Cognitive Impairments in Alcohol Use Disorder
Source: Brain Sci. 2022 Dec 26;13(1):45. doi: 10.3390/brainsci13010045 (PMC9856140; doi:10.3390/brainsci13010045)
Supplement: Supplementary file 1 [file brainsci-13-00045-s001.zip › brainsci-1995860-supplementary.pdf]

Supplementary Materials

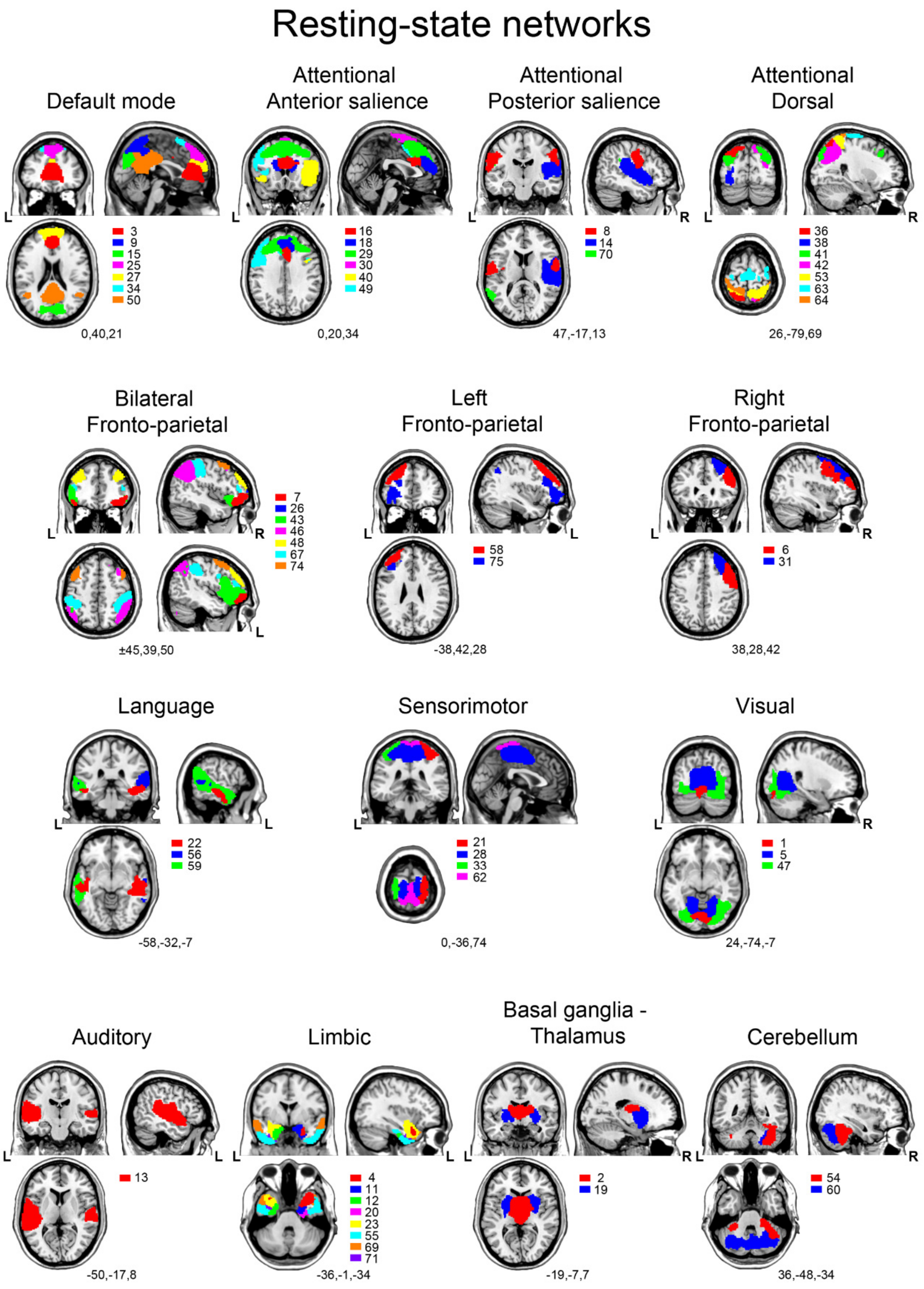

Figure S1. Resting-state network.

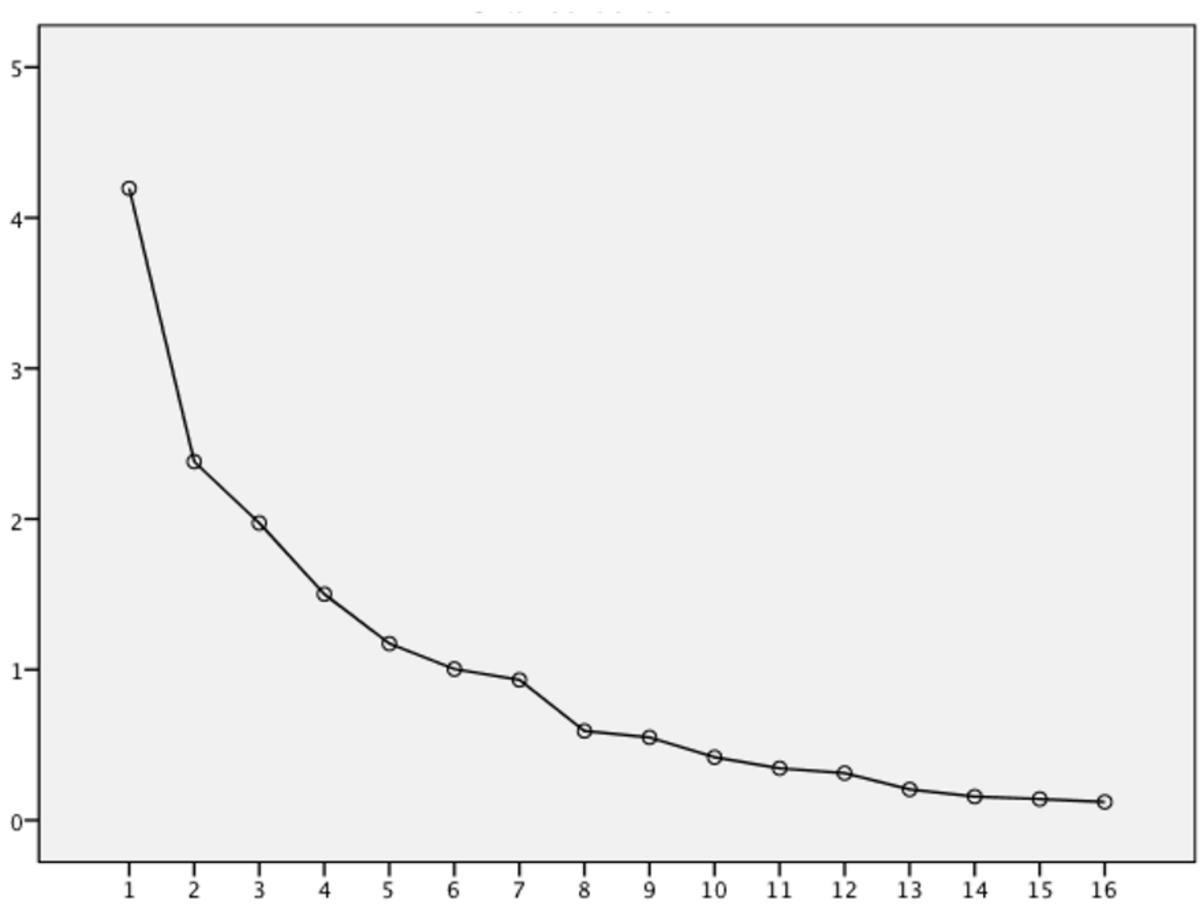

Figure S2. Scree plot of the principal component analysis performed on the 15 scores of the Brief Neuropsychological Examination (ENB2).

Table S1. Correlation Matrix.

	DS	IR	DR	IM-10	IM-30	TMT-A	TMT-B	TT	PF	AVR	CE	OF	CD	SD	CLD	PA
DS	1.000	.211	.100	.303	.011	-.171	-.188	-.300	.180	.152	.394	.157	.140	-.136	-.140	-.033
IR	.211	1.000	.787	.344	.171	-.369	-.448	.139	.088	.245	.118	.448	.151	.447	.365	.173
DR	.100	.787	1.000	.301	-.005	-.224	-.450	.344	.083	.151	.219	.333	.161	.363	.301	.047
IM-10	.303	.344	.301	1.000	.396	-.390	-.250	-.165	.089	.038	-.028	.252	-.002	.121	.089	.076
IM-30	.011	.171	-.005	.396	1.000	-.343	.100	-.084	.133	.008	-.170	.274	-.146	.299	.176	.279
TMT-A	-.171	-.369	-.224	-.390	-.343	1.000	.466	-.085	.119	-.104	-.024	-.525	-.104	-.152	-.283	-.005
TMT-B	-.188	-.448	-.450	-.250	.100	.466	1.000	-.164	.107	-.184	-.353	-.391	-.397	-.072	-.178	.092
TT	-.300	.139	.344	-.165	-.084	-.085	-.164	1.000	-.512	-.117	.226	.231	.037	.292	.087	-.044
PF	.180	.088	.083	.089	.133	.119	.107	-.512	1.000	.013	-.087	-.046	.106	-.080	.146	.107
AVR	.152	.245	.151	.038	.008	-.104	-.184	-.117	.013	1.000	.055	.207	.280	.127	.066	.295
CE	.394	.118	.219	-.028	-.170	-.024	-.353	.226	-.087	.055	1.000	.159	.403	.093	-.078	-.086
OF	.157	.448	.333	.252	.274	-.525	-.391	.231	-.046	.207	.159	1.000	.359	.479	.512	.353
CD	.140	.151	.161	-.002	-.146	-.104	-.397	.037	.106	.280	.403	.359	1.000	-.124	.096	-.086
SD	-.136	.447	.363	.121	.299	-.152	-.072	.292	-.080	.127	.093	.479	-.124	1.000	.469	.629
CLD	-.140	.365	.301	.089	.176	-.283	-.178	.087	.146	.066	-.078	.512	.096	.469	1.000	.576
PA	-.033	.173	.047	.076	.279	-.005	.092	-.044	.107	.295	-.086	.353	-.086	.629	.576	1.000

The table reports the cross-correlation coefficients among the 15 tests of the Brief Neuropsychological Examination (ENB2). DS = Digit span, IR = Immediate recall, DR= Delayed recall, IM-10 = Interference memory 10", IM-30 = Interference memory 30", TMT-A = Trial Making test A, TMT-B = Trial Making test B, TT = Token test, PF = Phonemic fluency, AVR = Abstract verbal reasoning, CE = cognitive estimation, OF = Overlapping figures, CD = copy drawing, SD = Spontaneous drawing, CLD = Clock drawing, PA = Praxis abilities.

Table S2. Communalities.

	Communalities
Digit span	.795
Immediate recall	.823
Delayed recall	.906
Interference memory 10′′	.682
Interference memory 30′′	.656
Trial Making test A	.792
Trial Making test B	.702
Token test	.809
Phonemic fluency	.754
Abstract verbal reasoning	.411
Cognitive estimation	.755
Overlapping figures	.752
Copy drawing	.743
Spontaneous drawing	.835
Clock drawing	.712
Praxis abilities	.856

For each variable of the Brief Neuropsychological Examination (ENB2), the table shows the proportion of variance explained by the retained components after the Varimax rotation.

Table S3. Rotated component matrix.

	Component					
	1	2	3	4	5	6
Digit span	**.916**					
Immediate recall	**.791**	.334				
Delayed recall	**.619**					-.387
Interference memory 10′′		**.936**				
Interference Memory 30′′		**.823**				
Trial Making test A			**-.779**	-.345		
Trial Making test B			**.711**			
Token test	.338		**.686**			
Phonemic fluency				**.839**		
Abstract verbal reasoning		-.466		**-.592**		
Cognitive estimation	.472		.439	**.496**		
Overlapping figures	.352			**.458**		
Copy drawing					**.844**	
Spontaneous drawing					**-.830**	
Clock drawing						**.791**
Praxis abilities				.315		**.716**

The table shows the correlations between the ENB variables and the estimated components after the Varimax rotation. Correlation coefficients < 0.3 are not reported.

Table S4. Peak activations of default-mode and executive networks.

Network	IC	H	Region	AT	x	y	z	K	t
**DMN**	3	Left	Anterior cingulate cortex		-6	34	10	3973	26.67
		Right	Anterior cingulate cortex		10	40	10		25.93
	9	Right	Precuneus	5L	2	-60	70	3861	21.58
		Left	Precuneus		-8	-58	58		21.28
	15	Right	Precuneus	7P	10	-76	48	4560	24.86
		Left	Precuneus	7P	-4	-82	48		19.54
	25	Left	Superior Medial Gyrus		-6	40	56	2209	18.86
		Right	Superior Medial Gyrus		14	52	42		17.84
	27	Right	Superior Medial Gyrus	Fp1	8	64	16	2973	18.35
		Left	Superior Medial Gyrus	Fp2	-4	64	14		14.69
	34	Left	Superior Frontal Gyrus		-18	28	58	2368	19.66
		Left	Posterior-Medial Frontal		0	18	68		17.92
	50	Left	Posterior cingulate cortex		-6	-44	10		23.49
		Right	Angular Gyrus		44	-62	36		18.27
		Left	Angular Gyrus	hIP1	-38	-60	42	1372	24.49
**Network**	**IC**	**H**	**Region**	**AT**	**x**	**y**	**z**	**K**	**t**
**Executive control-bilateral**	7	Right	IFG pars Orbitalis		40	42	-10	1556	19.84
		Left	IFG pars Orbitalis		-46	42	-14	489	18.28
	26	Right	Superior Medial Gyrus	Fp1	8	70	8	3018	17
		Right	Superior Frontal Gyrus	Fp1	16	72	8		16.41
		Left	Superior Frontal Gyrus	Fp1	-14	70	12		15.96
	43	Left	IFG pars Orbitalis		-46	24	-8	4150	24.16
		Left	Anterior insula		-34	20	-10		17.21
		Right	IFG pars Orbitalis		48	30	-4	713	16.45
		Left	SupraMarginal Gyrus	PFm	-54	-48	32	284	14.25
		Left	Posterior-Medial Frontal		-8	14	68	9	10.64
	46	Right	Angular Gyrus		50	-52	36	3550	31.93
		Right	Middle Frontal Gyrus		38	24	44	190	15.92
		Right	Middle cingulate cortex		8	-38	36	149	14.84
		Left	Middle cingulate cortex		0	-20	36	4	10.29
	48	Left	Middle Frontal Gyrus		-36	38	30	1965	19.24
		Left	Superior Frontal Gyrus	Fp1	-30	64	6		11.67
		Right	Middle Frontal Gyrus		36	44	30	1891	17.57
		Right	Superior Frontal Gyrus	Fp1	26	66	4		14.27
	67	Right	IFG pars Opercularis	44	52	14	20	124	13.67

		Right	Middle Frontal Gyrus		46	42	8	91	14.82
		Left	Middle Frontal Gyrus		-22	10	56	45	12.02
	74	Left	Middle Frontal Gyrus		-40	22	54	2098	20.14
		Left	Superior Frontal Gyrus		-26	-10	74		16.38
		Right	Superior Frontal Gyrus		24	-4	76	1333	17.28
		Right	Middle Frontal Gyrus		42	18	54		12.97
		Right	Superior Parietal Lobule	7PC	24	-50	66	52	11.26
**Network**	**IC**	**H**	**Region**	**AT**	**x**	**y**	**z**	**K**	**t**
**Executive control-left**	58	Left	Middle Frontal Gyrus		-22	34	40	4109	22.38
		Left	Superior Frontal Gyrus		-18	28	60		21.48
	75	Left	Middle Orbital Gyrus		-32	52	-14	2340	17.84
		Left	Middle Frontal Gyrus		-40	46	2		15.64
		Left	Superior Frontal Gyrus		-22	42	22		12.67
		Left	Angular Gyrus	hIP3	-36	-60	42	92	13.86
**Network**	**IC**	**H**	**Region**	**AT**	**x**	**y**	**z**	**K**	**t**
**Executive control-right**	6	Right	IFG pars Triangularis	45	48	26	22	4317	21.84
		Right	Middle Frontal Gyrus		44	16	56		21.57
	31	Right	Middle Frontal Gyrus		42	46	28	4193	22.28

Peak activations of the independent components belonging to default-mode and executive networks.

IC: independent component; H: hemisphere; AT: anatomy toolbox [1]; L: left; R: right; Fp2: medial frontopolar area 2; hIP: human intraparietal; IFG: inferior frontal gyrus; K: cluster extent in number of voxels (2x2x2 mm^3).

Table S5. Peak activations of attentional networks.

Network	IC	H	Region	AT	x	y	z	K	t
**Anterior salience**	16	Left	Anterior cingulate cortex		-2	28	24	2334	17.18
	18	Right	Anterior cingulate cortex		12	44	16	4996	20.34
		Right	Superior Medial Gyrus		4	54	18		16.09
		Right	Caudate Nucleus		14	18	14		14.57
	29	Right	Middle cingulate cortex		4	28	34	9668	19.44
		Left	Middle cingulate cortex		-4	28	34		19.29
		Left	Superior Frontal Gyrus		-14	14	56		17.55
		Right	Superior Frontal Gyrus		18	44	38		16.78
	30	Left	Posterior-Medial Frontal		-2	14	70	2356	22.17
		Right	Posterior-Medial Frontal		8	-6	70		19.52
	40	Right	IFG pars Orbitalis		42	34	-8	4992	20.74
		Right	Anterior insula		34	26	0		18.22
		Left	IFG pars Orbitalis		-50	22	-4	219	12.51
		Left	Anterior insula		-36	14	6	14	11.12
	49	Left	IFG pars Triangularis	45	-52	32	20	4341	18.79
		Left	Precentral Gyrus		-54	0	50		18.7
		Left	Posterior-Medial Frontal		-8	12	48	819	16.89
		Left	Anterior insula		-36	18	8	229	15.5
		Right	Precentral Gyrus		38	2	48		11.82
		Right	IFG pars Opercularis		46	14	28	95	11.72
**Network**	**IC**	**H**	**Region**	**AT**	**x**	**y**	**z**	**K**	**t**
**Posterior salience**	8	Right	Rolandic Operculum		58	2	16		18.63
		Left	Rolandic Operculum	OP4	-56	-8	12		18.08
		Left	Inferior Parietal Lobule	2	-54	-20	38		14.59
	14	Right	Superior Temporal Gyrus		52	-2	2	6453	24.15
		Right	Rolandic Operculum	OP4	58	-10	10		22.17
		Right	Middle insula		42	14	2		17.47
	70	Left	Angular Gyrus	PGp	-56	-62	24	3668	22.72
		Left	SupraMarginal Gyrus	PFop	-64	-28	24		20.93
		Right	SupraMarginal Gyrus	PFcm	58	-30	28	752	16.02
		Left	Middle insula		-46	8	-4	33	11.71
**Network**	**IC**	**H**	**Region**	**AT**	**x**	**y**	**z**	**K**	**t**
**Dorsal attentional**	36	Left	Angular Gyrus	PFm	-44	-58	40	5125	26.35

		Left	Superior Parietal Lobule	7P	-14	-76	58		18.42
		Right	Superior Parietal Lobule	7A	32	-70	56	277	17.67
		Left	Precentral Gyrus		-44	4	32	22	11.72
	38	Left	Fusiform Gyrus	FG1	-30	-62	-14	5172	22.02
		Left	Inferior Temporal Gyrus	FG4	-46	-60	-10		20.07
	41	Left	Angular Gyrus	PGp	-44	-74	44		18.85
		Left	Superior Parietal Lobule	7P	-20	-82	52		18.02
		Right	Angular Gyrus	PGp	46	-72	36	2112	25.69
		Right	Superior Parietal Lobule		26	-82	48		14.31
		Right	Middle Frontal Gyrus		24	28	40	139	15.74
		Left	Middle Frontal Gyrus		-24	18	46	97	13.08
	42	Right	Middle Occipital Gyrus		32	-72	22	4322	19.71
		Right	Superior Occipital Gyrus		22	-64	44		19.35
		Right	Superior Parietal Lobule	7A	26	-64	60		19.13
		Left	Superior Parietal Lobule	7A	-14	-72	54	854	17.8
	53	Right	Superior Parietal Lobule		40	-60	60	2223	17.45
		Left	Superior Parietal Lobule	7A	-18	-64	70	828	17.02
		Left	Inferior Parietal Lobule		-44	-52	60		13.81
	63	Right	Paracentral Lobule		4	-26	80	2970	22.9
		Left	Paracentral Lobule		-8	-34	82		21.53
		Right	Precentral Gyrus		28	-16	76		17.33
		Left	Precentral Gyrus		-28	-24	76		16.69
	64	Left	Superior Parietal Lobule	5L	-18	-56	74	1624	20.33
		Right	Superior Parietal Lobule	7PC	22	-50	76	292	15.72

Peak activations of the independent components belonging to anterior salience, posterior salience and dorsal attentional networks. IC: independent component; H: hemisphere; AT: anatomy toolbox [1]; L: left; R: right; IFG: inferior frontal gyrus; OP: parietal operculum; FG: fusiform gyrus; K: cluster extent in number of voxels (2x2x2 mm^3).

Table S6. Peak activations of language, sensorimotor, visual and auditory networks.

Network	IC	H	Region	AT	x	y	z	K	t
**Language**	22	Right	Middle Temporal Gy-rus		56	-36	-4	2756	16.38
		Right	Superior Temporal Gy-rus		44	-4	-12		15.06
		Left	Middle Temporal Gy-rus		-50	-18	-20	1226	17.35
		Left	Inferior Temporal Gy-rus		-58	-6	-30		11.66
	56	Right	Middle Temporal Gy-rus		48	-60	12	4439	25.5
		Right	Superior Temporal Gy-rus		62	-26	2		23.54
		Right	Angular Gyrus	PGp	54	-68	34		15.3
		Left	Middle Temporal Gy-rus		-56	-54	6	111	12.47
	59	Left	Middle Temporal Gy-rus	PGa	-56	-58	22	5259	22.01
		Left	Inferior Temporal Gy-rus		-52	2	-36		18.85
		Left	Middle Frontal Gyrus		-36	8	52	17	11.12
**Network**	**IC**	**H**	**Region**	**AT**	**x**	**y**	**z**	**K**	**t**
**Sensorimo-tor**	21	Right	Precentral Gyrus	4p	36	-20	48	4324	17.42
		Right	Postcentral Gyrus	3b	42	-28	48		17.11
		Left	Postcentral Gyrus		-48	-18	60	14	10.61
	28	Left	Precentral Gyrus	4a	-32	-28	58	11864	23.15
		Left	Middle cingulate cortex		-8	-10	44		21.94
		Right	Paracentral Lobule	4a	8	-34	54		21.25
	33	Left	Precentral Gyrus		-40	-18	54	4380	21.01
		Left	Postcentral Gyrus	4p	-36	-28	56		18.58
		Left	Inferior Parietal Lobule	1	-56	-24	46		15.98
		Right	Precentral Gyrus		42	-12	66	37	12.43
	62	Right	Paracentral Lobule		4	-24	78	2275	21.07
		Left	Precuneus	5M	-6	-52	74		19.82
		Right	Precuneus	5L	8	-52	76		17.07
**Network**	**IC**	**H**	**Region**	**AT**	**x**	**y**	**z**	**K**	**t**
**Visual**	1	Left	Lingual Gyrus		-2	-78	-10	1815	16.97
		Right	Lingual Gyrus	hOc3v [V3v]	20	-86	-14		14.5
		Left	Fusiform Gyrus	hOc4v [V4v]	-28	-78	-16		10.51
	5	Right	Calcarine Gyrus	hOc2 [V2]	14	-60	12	8743	28.1

		Right	Lingual Gyrus	hOc1 [V1]	12	-78	0		23.73
		Left	Calcarine Gyrus	hOc3d [V3d]	-6	-72	16		21.61
		Left	Lingual Gyrus	hOc1 [V1]	0	-76	2		20.95
	47	Right	Inferior Temporal Gy-rus		46	-60	-12	6872	20.44
		Right	Inferior Occipital Gyrus	FG1	38	-70	-10		19.34
		Right	Fusiform Gyrus	FG2	44	-58	-22		18.87
		Right	Middle Occipital Gyrus		32	-82	18		17.4
**Network**	**IC**	**H**	**Region**	**AT**	**x**	**y**	**z**	**K**	**t**
**Auditory**	13	Left	Superior Temporal Gy-rus	TE 3	-64	-26	12		21.08
		Left	Superior Temporal Gy-rus	TE 1.1	-42	-28	8		19.53
		Right	Superior Temporal Gy-rus	TE 1.2	54	-2	0		15.1

Peak activations of the independent components belonging to language, sensorimotor, visual and auditory networks. IC: independent component; H: hemisphere; AT: anatomy toolbox [1]; L: left; R: right; hOC: human occipital complex; FG: fusiform gyrus; K: cluster extent in number of voxels (2x2x2 mm^3).

Table S7. Peak activations of limbic, basal ganglia and cerebellar networks.

Network	IC	H	Region	AT	x	y	z	K	t
**Limbic**	4	Right	ParaHippocampal Gy-rus	Fo3	22	12	-28	1906	20.16
		Right	Temporal Pole		32	12	-26		19.45
		Right	Amygdala	LB	22	4	-26		18.93
	11	Right	ParaHippocampal Gy-rus		18	4	-26	1151	18.15
	12	Left	ParaHippocampal Gy-rus	Entorhinal Cor-tex	-20	0	-32	1508	14.3
	20	Right	Hippocampus	Subiculum	12	-8	-26	1467	19.69
	23	Left	Amygdala		-30	2	-20	2247	19.25
		Left	Temporal Pole		-22	8	-30		16.26
	55	Right	Inferior Temporal Gy-rus		48	0	-40	1672	20.3
		Right	Fusiform Gyrus		40	-14	-36		18.42
		Left	Inferior Temporal Gy-rus		-40	-10	-38	1500	20.79
		Left	Fusiform Gyrus		-30	-16	-32		18.21
	69	Right	Temporal Pole		38	22	-28	2055	21.22
		Right	Medial Temporal Pole		38	18	-40		19.6
		Right	IFG pars Orbitalis		46	24	-18		14.23
		Left	Medial Temporal Pole		-48	14	-32	1840	21.52
		Left	Temporal Pole		-46	18	-20		16.58
	71	Left	Fusiform Gyrus		-32	-4	-46	1242	17.13
		Left	ParaHippocampal Gy-rus	Entorhinal Cor-tex	-20	-8	-36		15.36
		Left	Medial Temporal Pole		-24	8	-38		12.67
**Network**	**IC**	**H**	**Region**	**AT**	**x**	**y**	**z**	**K**	**t**
**Basal gan-glia**	2	Left	Caudate Nucleus		-8	6	4	4945	29.92
		Left	Thalamus		-8	-20	16		25.92
		Right	Thalamus		6	-14	16		24.71
	19	Right	Insula Lobe		36	12	2	3109	20.1
		Right	Putamen		26	2	8		19.31
		Right	Caudate Nucleus		18	14	12		17.27
		Left	Putamen		-24	0	6	2654	18.81
**Network**	**IC**	**H**	**Region**	**AT**	**x**	**y**	**z**	**K**	**t**
**Cerebellum**	54	Right	Cerebellum (VIII)	Lobule VIIa crusII	36	-48	-48	2400	19.29
		Right	Cerebellum (X)		24	-32	-42		18.45
		Right	Cerebellum (VI)	Lobule VI	32	-44	-38		17.88
		Right	Cerebellum (IV-V)	Lobule V	30	-38	-28		17.04

		Right	Cerebellum	Lobule VIIIb	20	-38	-52		14.93
		Right	Cerebellum (Crus 1)	Lobule VIIa crusI	38	-56	-34		14.33
		Left	Cerebellum (X)		-28	-36	-42	556	19.14
	60	Right	Cerebellum (VI)	Lobule VI	30	-58	-26	8460	26.08
		Right	Cerebellum (VI)	Lobule VIIa crusI	32	-68	-26		22.05
		Right	Cerebellum (Crus 1)	Lobule VIIa crusI	26	-72	-34		20.71
		Left	Cerebellum (Crus 1)	Lobule VIIa crusI	-40	-64	-30		20.52
		Left	Cerebellum (Crus 2)	Lobule VIIa crusI	-6	-82	-30		18.62

Peak activations of the independent components belonging to limbic, basal ganglia and cerebellar networks.
IC: independent component; H: hemisphere; AT: anatomy toolbox [1]; L: left; R: right; Fo3: medial orbital sulcus; LB: latero-basal amygdala nuclei; IFG: inferior frontal gyrus; K: cluster extent in number of voxels (2x2x2 mm^3).

Table S8. Total variance explained.

Component	A. Initial eigenvalues			B. Rotation Sums of Squared Loadings		
	Total	% of Variance	Cumulative %	Total	% of Variance	Cumulative %
1	4.195	26.221	26.221	2.441	15.257	15.257
2	2.381	14.881	41.102	2.441	15.256	30.513
3	1.973	12.333	53.435	2.127	13.293	43.805
4	1.501	9.383	62.818	1.908	11.922	55.727
5	1.173	7.330	70.148	1.819	11.370	67.097
6	1.004	6.274	76.422	1.492	9.324	76.422
7	.932	5.824	82.246			
8	.592	3.701	85.946			
9	.550	3.437	89.383			
10	.418	2.613	91.996			
11	.344	2.153	94.149			
12	.313	1.954	96.103			
13	.204	1.278	97.380			
14	.157	.982	98.362			
15	.141	.878	99.241			
16	.121	.759	100.000			

The table reports the distribution of the variance (eigenvalues, percentage of total variance accounted by each component, and the relative cumulative percentage) before (A) and after (B) a Varimax rotation. Based on the Kaiser-Guttman criterion (eigenvalue > 1), we retained six components explaining 76.42% of the total variance.

Table S9. Principal component analysis of neuro-cognitive data.

Principal component	Proportion of variance explained (cumulative %)	ENB2 tests	Loading coefficient
#1: Visuo-constructional abilities	15.26%	Ideative and ideomotor praxis test	0.922
		Spontaneous drawing	0.755
		Clock drawing test	0.651
#2: Verbal learning	15.25% (30.51%)	Logical story: delayed recall	0.940
		Logical story: immediate recall	0.820
#3: Basic-level executive functions	13.29% (43.80%)	TMT-A	-0.787
		Interference memory test 10″	0.716
		Interference memory test 30″	0.716
#4: High-level executive functions	11.92% (55.72%)	Copy drawing	0.920
		TMT-B	-0.573
		Overlapping pictures test	0.486
		Abstract verbal reasoning	0.394
#5: Language	11.37% (67.09%)	Phonemic fluency	0.862
		Token test	-0.828
#6: Estimation-related processes	9.32% (76.42%)	Digit span	0.826
		Cognitive estimation	0.626

The results of a principal component analysis performed on the 15 ENB2 scores obtained by AUD patients and healthy controls. From left to right, columns report: a) the first 6 components (eigenvalue>1) explaining 76.42% of the total variance of participants' performance in the 15 ENB2 tests; b) the relative contribution of each component, in terms of specific and cumulative proportion of variance explained; c, d) the single ENB2 tests contributing to each component, and their loading coefficients.

References

1. Eickhoff, S.B.; Stephan, K.E.; Mohlberg, H.; Grefkes, C.; Fink, G.R.; Amunts, K.; Zilles, K. A new SPM toolbox for combining probabilistic cytoarchitectonic maps and functional imaging data. *Neuroimage* **2005**, *25*, 1325–1335. https://doi.org/10.1016/j.neuroimage.2004.12.034
